# Type 2 diabetes mellitus and In-hospital Major Adverse Cardiac and Cerebrovascular Events (MACCEs) and postoperative complications among patients undergoing on-pump isolated coronary artery bypass surgery in Northeastern Iran

**DOI:** 10.1186/s12872-023-03163-5

**Published:** 2023-03-11

**Authors:** Mahin Nomali, Aryan Ayati, Amirhossein Tayebi, Mohammad Eghbal Heidari, Keyvan Moghaddam, Soheil Mosallami, Gholamali Riahinokandeh, Mahdis Nomali, Gholamreza Roshandel

**Affiliations:** 1 Department of Epidemiology and Biostatistics, School of Public Health, Tehran University of Medical Sciences, Tehran, Iran; 2 Endocrinology and Metabolism Research Center, Endocrinology and Metabolism Clinical Sciences Institute, Tehran University of Medical Sciences, Tehran, Iran; 3 Cardiovascular Diseases Research Institute, Tehran University of Medical Sciences, Tehran, Iran; 4 Clinical Research Development Unit (CRDU), Shahid Rajaei Educational & Medical Center, Alborz University of Medical Sciences, Karaj, Iran; 5 Student Research Center, School of Nursing and Midwifery, Tehran University of Medical Sciences, Tehran, Iran; 6 Kordkuy Amiralmomenin Hospital, Golestan University of Medical Sciences, Gorgan, Iran; 7 Department of Surgery, School of Medicine, Sayyad Shirazi Hospital, Kordkuy Amiralmomenin Hospital, Golestan University of Medical Sciences, Gorgan, Iran; 8 Alejalil Hospital, Golestan University of Medical Sciences, Gorgan, Iran; 9 Golestan Research Center of Gastroenterology and Hepatology, Golestan University of Medical Sciences, Gorgan, Iran

**Keywords:** Diabetes Mellitus, Type2, Coronary artery bypass, Major adverse cardiovascular events, Complications, Retrospective studies

## Abstract

**Background:**

Diabetes Mellitus (DM) is a rapidly growing disorder worldwide, especially in the Middle East. A higher incidence of coronary artery diseases requiring coronary artery bypass graft (CABG) surgery has been reported in patients with diabetes. We assessed the association between type 2 diabetes mellitus (T2DM) and in-hospital major adverse cardiac and cerebrovascular events (MACCEs) and postoperative complications among patients who underwent on-pump isolated CABG.

**Methods:**

In this retrospective cohort study, we used the data registered for CABG patients from two heart centers in the Golestan province (North of Iran) between 2007 and 2016. The study population included 1956 patients divided into two groups: 1062 non-diabetic patients and 894 patients with diabetes (fasting plasma glucose ≥126 mg/dl or using antidiabetic medications). The study outcome was in-hospital MACCEs, a composite outcome of myocardial infarction (MI), stroke and cardiovascular death, and postoperative complications, including postoperative arrhythmia, acute atrial fibrillation (AF), major bleeding (defined as reoperation due to bleeding), and acute kidney injury (AKI).

**Results:**

During the 10-year study period, 1956 adult patients with a mean (SD) age of 59.0 (9.60) years were included. After adjustment for age, gender, ethnicity, obesity, opium consumption, and smoking, diabetes was a predictor of postoperative arrhythmia (AOR 1.30, 95% CI 1.08–1.57; *P* = 0.006). While it was not a predictor of in-hospital MACCEs (AOR 1.35, 95% CI 0.86, 2.11; *P* = 0.188), AF (AOR 0.85, 95% CI 0.60–1.19; *P* = 0.340), major bleeding (AOR 0.80, 95% CI 0.50, 1.30; *P* = 0.636) or AKI (AOR 1.29, 95% CI 0.42, 3.96; P 0.656) after CABG surgery.

**Conclusion:**

Findings indicated that diabetes increased the risk of postoperative arrhythmia by 30%. However, we found similar in-hospital MACCEs, acute AF, major bleeding, and AKI following CABG surgery in both diabetic and non-diabetic patients.

**Supplementary Information:**

The online version contains supplementary material available at 10.1186/s12872-023-03163-5.

## Background

Type 2 Diabetes Mellitus (T2DM) is a multifaceted disorder affecting an increasing portion of the population worldwide [1, 2]. The Middle East, in particular, is one of the primary regions where the number of diabetic patients is rapidly increasing [3, 4]. Diabetic patients are associated with a 4.4-fold increased risk due to cardiovascular disorders [5].

While percutaneous interventions (PCI) have become a widespread treatment for coronary artery disease (CAD), coronary artery bypass graft surgery (CABG) remains the optimal strategy for patients with severe and multivessel CADs. In addition, due to the multivessel nature of CAD in patients with T2DM, various studies have suggested that CABG surgery can be superior to PCI in patients with diabetes and other major cardiovascular risk factors [6–8]. Therefore, CABG surgery is more common among patients with diabetes.

Diabetes can also affect the postoperative outcomes of CABG [9, 10], besides having a greater risk of CAD and CAD-related death [11]. Thus, recent studies have reported worse postoperative outcomes for CABG patients with DM [12–14].

The northeastern region of Iran is significant due to its unique ethnic profile. Unlike other areas of the country, a large population of Turkmen ethnicities resides in this region [15]. Several studies of the population of northeastern Iran and Turkmen ethnics have reported a higher prevalence of congenital heart disorders [16], obesity [17], metabolic syndrome [18], cardiovascular diseases [19], hypertension [20], and a high waist circumference [18] compared to other regions of the country. According to the Golestan Cohort Study data, a significantly lower diabetes awareness was reported in this region [21]. The precise reason for these discrepancies is unknown. However, genetic predispositions, higher rural populations, and socioeconomic and lifestyle differences are considered the main contributing factors to this region’s higher prevalence of cardiovascular risk factors [19, 22].

Furthermore, there has been little research on the relationship between diabetes and cardiovascular disorders in this region. As a result, we sought to investigate the effect of T2DM on in-hospital major adverse cardiac and cerebrovascular events (MACCEs) and postoperative complications, such as postoperative arrhythmia, acute AF, major bleeding, and acute kidney injury (AKI) in patients undergoing on-pump isolated CABG in Iran’s northeastern region. Understanding the effects of diabetes on the postoperative outcomes of CABG surgery can be essential for applying better treatment strategies for these patients, considering the unique characteristics of the population in this region.

## Methods

### Study design

This retrospective cohort study was performed in northeastern Iran (Golestan province, Iran). The study protocol was approved by the institutional review board (IRB) (approval ID 950505.06) and research ethics committee (REC) (ID IR.GOUMS.REC.1395.137) of Golestan University of Medical Sciences in 2016.

### Setting

In this study, we used the data registry of the characteristics of patients who underwent CABG surgery from 2007 to 2016 in Golestan province. Data were collected from two heart centers, including the Kordkuy heart center of Amiralmomenin hospital, affiliated with Golestan University of Medical Sciences (Gorgan, Iran), and the Shafa private heart center, which provides medical services to patients all over the province.

### Participants

Adult patients (> 18 years old) of both sexes who had isolated on-pump CABG procedures between 2007 and 2016 and had complete data on study exposure (status of DM) and covariates were included in the study analysis.

### Variables and measurement

Study variables were demographic and clinical, including past medical history, comorbidities, lipid profile, preoperative medications, clinical characteristics, and operation characteristics. The demographic variables included age, gender, ethnicity, obesity, opium consumption, and smoking status. Opium consumption and smoking status were self-reported data. We considered ethnicity as Turkmen and non-Turkmen ethnicities, the main ethnicities living in Golestan province. In order to define obesity, we calculated the body mass index (BMI) according to the weight and height of each patient. Then, according to the BMI categorization by the center for disease control and prevention (CDC), we considered a BMI of 30 or above as obese [23]. For past medical history, we retrieved the family history of cardiovascular diseases (CVDs) and previous myocardial infarction (MI), which were self-reported data. In addition, various comorbidities were assessed, including hyperlipidemia, hypertension (HTN), chronic obstructive pulmonary disease (COPD), and a lipid profile consisting of low-density lipoprotein (LDL), total cholesterol, and non-high-density lipoprotein (HDL) cholesterol. Non-HDL cholesterol refers to the total cholesterol value minus the HDL cholesterol value. Preoperative medications included Beta-blocker (BB), Statins, and Aspirin. Preoperative clinical characteristics were left ventricular ejection fraction (LVEF) by transthoracic echocardiography (TTE), diagnosis of three-vessel disease, and left main coronary artery (LMCA) stenosis by coronary artery angiography (CAG). Also, operation characteristics included emergency CABG, the left internal mammary artery (LIMA) graft, the number of grafts, cardiopulmonary bypass (CPB) time, and clamp time, which were recorded according to the surgeon’s and perfusionist’s report.

The study outcomes were in-hospital MACCEs (a composite outcome of MI, stroke, and cardiovascular death), postoperative arrhythmia, acute atrial fibrillation (AF), major bleeding, and AKI. Further interpretation and analysis regarding MACCE definitions are presented in the appendix.

- Myocardial infarction was defined as clinical evidence of acute myocardial ischemia and detection of a rise in cardiac troponin levels with at least one value above the 99th percentile upper limit and at least one of the following:
  - Symptoms of myocardial ischemia
  - New ischemic electrocardiogram changes
  - Development of pathological Q waves [24]
- Stroke was defined as a new focal neurological deficit that lasted more than 24 hours and was confirmed by imaging [25].
- Postoperative AF and arrhythmias were detected by electrocardiograms (ECGs) read by the attending cardiologists.
- Major postoperative bleeding was defined as significant bleeding requiring a reoperation [26].
- AKI was defined as an absolute increase in serum creatinine concentration of 0.3 mg/dL or greater [27, 28].
- Study exposure was T2DM, which was defined according to fasting plasma glucose (FPG) ≥126 mg/dl or using antidiabetic medications (i.e., Anti-diabetes oral tablets or Insulin).

### Operative Procedure

After general anesthesia, a median sternotomy and aortic-right atrial cannulation were performed to achieve a cardiopulmonary bypass [29]. The ascending aorta was occluded, and a cardioplegic solution (Thomas crystalloid cardioplegia solution) was perfused into the heart during CABG [30, 31]. Several infusions of the cardioplegic solution might be administered if an electrical activity or prolonged ischemic time was observed. After completing distal anastomoses, the aortic cross-clamp was removed during myocardial revascularization. After reperfusion, partial occlusion clamps were used to complete the proximal anastomosis. The cross-clamp could still be used after the distal grafts have been conducted to facilitate the proximal grafts [32]. The left internal thoracic artery and greater saphenous vein were the most commonly used bypass grafts.

### Statistical analysis

First, we compared the patients` data across diabetes status. The normality assumption for continuous variables was checked graphically by a histogram plot and the Shapiro-Wilk statistical test. Because of the non-normal distribution of the study variables, they were reported as the median and interquartile range (IQR) and were compared using Mann-Whitney U tests. In order to compare categorical variables, Chi-square tests were used, and the data were reported as numbers and percentages. We used univariate logistic regression analysis to evaluate the association between diabetes and in-hospital outcomes. Then, we used the change-in-estimate (CIE) criterion with a cut-off of more than 10% to detect the probable confounders of this association. Multiple logistic regression analysis was used to adjust for confounders identified by the CIE criterion. Both crude and adjusted ORs were reported for the association between diabetes and in-hospital outcomes with a 95% confidence interval (CI). Data analyses were performed by the statistical package STATA/IC version 14.2 (Stata Corp LP College Station, TX, USA).

## Results

**Fig. 1:**
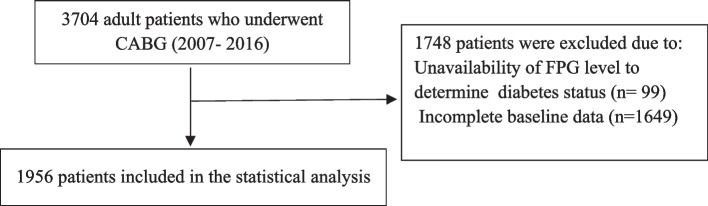
Study population diagram

During the 10-year study from 2007 to 2016, demographic and clinical characteristics of 3704 patients who underwent on-pump isolated CABG surgery in the Golestan province were registered. Data related to 1748 patients were excluded due to incomplete data regarding study exposure and baseline data. Thus, the study population included 1956 patients (Fig. 1) with a mean (SD) age of 59.0 (9.60) years. According to the definitions, 1062 patients were non-diabetics, and 894 had diabetes.

### Baseline Characteristics

The patients’ data are demonstrated in Table 1. According to this table, patients with DM were younger, and 44% were female, significantly different from patients without diabetes. Opium consumption and smoking were lower among patients with diabetes. The two groups were similar regarding family history of CVDs, prior MI, and comorbidities. Although there were no differences in lipid profiles between the two groups, LDL, total cholesterol, and non-HDL cholesterol levels were higher than normal values. The frequency of Beta-blocker, Statin and Aspirin consumption, three-vessel disease, and the number of grafts > 3 were higher among patients with diabetes compared to non-diabetics. The proportion of patients with DM with 40% LVEF was higher than in the non-diabetic group (20% vs. 17%), but this difference was not statistically significant (*P* = 0.061). However, CPB time and clamp time were similar among the two groups (Table 1).

**Table 1:** Baseline characteristics of patients with and without diabetes mellitus undergoing isolated on-pump CABG surgery

Variables	Without DM (***n*** = 1062)	With DM (***n*** = 894)	***P*** value
Age (year) [Median (IQR)]	59 (53–66)	58 (53–64)	0.040*
< 65 years, n (%)	748 (70.0)	684 (76.5)	0.003*
≥ 65 years, n (%)	314 (30.0)	210 (23.5)	
Gender, n (%)			< 0.001*
Male	744 (71.0)	498 (56.0)	
Female	318 (29.0)	396 (44.0)	
Ethnicity, n (%)			0.660
Turkmen	73 (7.0)	57 (6.0)	
Non- Turkmen	989 (93.0)	837 (94.0)	
Obesity, n (%)			0.071
Yes	277 (27.0)	266 (30.0)	
No	785 (73.0)	628 (70.0)	
Opium consumption, n (%)			< 0.001*
Ever users	347 (33.0)	197 (22.0)	
Never users	715 (67.0)	697 (78.0)	
Smoking status, n (%)			0.001*
Ever users	196 (18.5)	115 (13.0)	
Never users	866 (81.5)	779 (87.0)	
**Past Medical history**			
Family history of CVDs, n (%)			0.117
Yes	781 (74.0)	685 (77.0)	
No	281 (26.0)	209 (23.0)	
Prior MI, n (%)			0.811
Yes	44 (4.0)	39 (4.0)	
No	1018 (96.0)	855 (96.0)	
**Comorbidities**			
Hyperlipidemia, n (%)			0.310
Yes	617 (58.0)	499 (56.0)	
No	445 (42.0)	395 (44.0)	
HTN, n (%)			0.132
Yes	679 (64.0)	542 (61.0)	
No	383 (36.0)	352 (40.0)	
COPD, n (%)			0.606
Yes	39 (4.0)	29 (3.0)	
No	1023 (96.0)	865 (97.0)	
**Lipid profile**			
LDL (mg/dl) [Median (IQR)]	146 (122–180)	149 (128–184)	0.230
Total cholesterol (mg/dl) [Median (IQR)]	215 (185–286)	215 (186–280)	0.512
Non-HDL (mg/dl) [Median (IQR)]	173 (141–244)	175 (143–242)	0.750
**Preoperative Medications**			
β-blockers, n (%)			< 0.001*
Yes	466 (44.0)	467 (52.0)	
No	596 (56.0)	427 (48.0)	
Statins, n (%)			0.001*
Yes	755 (72.0)	693 (78.0)	
No	307 (28.0)	201 (22.0)	
Aspirin, n (%)			< 0.001*
Yes	862 (82.0)	782 (88.0)	
No	200 (18.0)	112 (12.0)	
**Preoperative clinical characteristics**			
LVEF(%)			0.061
≤ 40%	180 (17.0)	181 (20.0)	
> 40%	882 (83.0)	713 (80.0)	
Three vessel disease, n (%)			0.020*
Yes	706 (66.5)	638 (71.0)	
No	356 (33.5)	256 (29.0)	
LMCA stenosis, n (%)			0.153
Yes	85 (8.0)	88 (10.0)	
No	977 (92.0)	806 (90.0)	
**Operation characteristics**			
Emergency CABG, n (%)			0.082
Yes	21 (2.0)	9 (1.0)	
No	1041 (98.0)	885 (99.0)	
LIMA graft, n (%)			0.147
Yes	1031 (98.0)	877 (98.0)	
No	31 (2.0)	17 (2.0)	
Number of grafts, n (%)			0.002*
≤ 3 grafts	606 (57.0)	448 (50.0)	
> 3 grafts	456 (43.0)	446 (50.0)	
CPB time (min) [Median (IQR)]	110 (59–130)	110 (57–128)	0.279
Clamp time (min) [Median (IQR)]	38 (30–48)	38 (31–50)	0.627

*DM* Diabetes Mellitus, *P value* Probability value, *IQR* Interquartile range, *CVDs* Cardiovascular diseases, *MI* Myocardial infarction, *HTN* Hypertension, *COPD* Chronic obstructive pulmonary disease, *AF* Atrial fibrillation, *LDL* Low-density lipoprotein, *HDL* High-density lipoprotein, *LVEF* Left ventricular ejection fraction, *LMCA* Left main coronary artery, *LIMA* Left internal mammary artery, *CPB* Cardiopulmonary bypass

### Postoperative Data

In-hospital adverse events occurred in 5% (n/*N* = 44/894) of patients with diabetes and 4% (n/*N* = 39/1062) of patients without diabetes (*P* = 0.172). According to the crude analysis, diabetes was not significantly associated with in-hospital MACCEs (Crude OR 1.36, 95%CI 0.87, 2.11; *P* = 0.174). Furthermore, after adjustment for age, gender, ethnicity, obesity, opium consumption, and smoking, diabetes was not a predictor of major adverse events following CABG surgery (adjusted OR 1.35, 95% CI 0.86, 2.11; *P* = 0.188). (Table 2).

**Table 2:** Comparison of postoperative complications between patients with and without diabetes mellitus undergoing isolated on-pump CABG surgery

In- hospital outcomes	Diabetes Group		Crude OR (95% CI)	***P***_value	Adjusted OR (95% CI)	***P***_value
	Without DM (***n*** = 1062)	With DM (***n*** = 894)				
**MACCEs**, n (%)			1.36 (0.87, 2.11)	0.174	1.35 (0.86, 2.11)	0.188 **
Yes	39 (4.0)	44 (5.0)				
No	1023 (96.0)	850 (95.0)				
**Post-op arrhythmia**, n (%)			1.20 (0.99, 1.43)	0.052	1.30 (1.08, 1.57)	0.006*^,^***
Yes	457 (44.0)	424 (47.0)				
No	605 (57.0)	470 (53.0)				
**Acute AF**, n (%)			0.80 (0.60, 1.1)	0.158	0.85 (0.60, 1.19)	0.340**
Yes	92 (9.0)	62 (7.0)				
No	970 (91.0)	832 (93.0)				
**Major bleeding**, n (%)			0.75 (0.47, 1.18)	0.216	0.80 (0.50,1.30)	0.636****
Yes	50 (5.0)	32 (4.0)				
No	1012 (95.0)	862 (96.0)				
**Acute kidney injury**, n (%)			1.39 (0.46, 4.15)	0.556	1.29 (0.42, 3.96)	0.656**
Yes	6 (1.0)	7 (1.0)				
No	1056 (99.0)	887 (99.0)				

*OR* Odds ratio, *CI* Confidence Interval, *P_value* Probability value

*MACCEs* Major adverse cardiac and cerebrovascular events (i.e., composite of MI, stroke and cardiovascular death), *AF* Atrial fibrillation, Post-op Postoperative

*Statistically significant (*P* < 0.05)

**Adjusted by age ≥ 65 years, gender, ethnicity, obesity, opium consumption, and smoking status

***Adjusted by age ≥ 65 years, gender, ethnicity, obesity, opium consumption, smoking status, preoperative Aspirin

****Adjusted by age ≥ 65 years, gender, ethnicity, obesity, opium consumption, smoking status, number of grafts, LIMA graft, CPB time, and clamp time

**Fig. 2:**
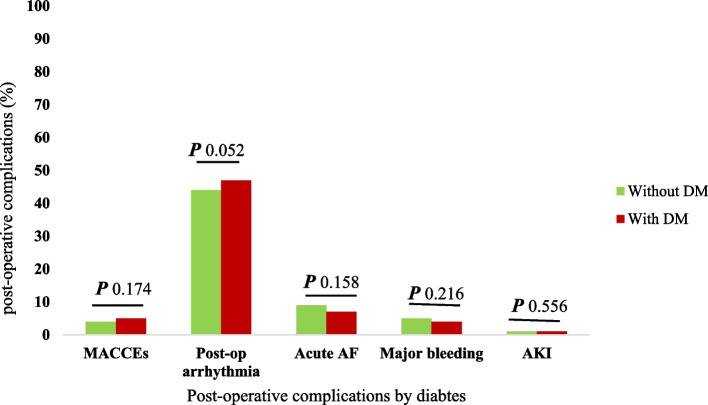
Postoperative complications by diabetes. MACCEs: Major adverse cardiac and cerebrovascular events (i.e., composite of MI, stroke and cardiovascular death). Post-op: Postoperative. AF: Atrial fibrillation. DM: Diabetes. AKI: Acute kidney injury. P: Probability value (i.e. *P* < 0.05 was considered as statistically significant)

The distribution of postoperative complications in patients with and without diabetes is demonstrated in Fig. 2. Despite a higher proportion of in-hospital MACCEs and postoperative arrhythmia in patients with diabetes compared to non-diabetics, these differences were not statistically significant (P 0.174 and P 0.052, respectively).

Table 2 compares postoperative complications between patients with and without diabetes mellitus undergoing isolated on-pump CABG surgery and indicates the results of the crude and adjusted analyses. According to this table, patients with diabetes experienced a higher proportion of in-hospital MACCEs compared to those without diabetes (5% vs. 4%, respectively). However, the crude analysis indicated no significant association between diabetes and in-hospital MACCEs (crude OR 1.36, 95% CI 0.87, 2.11; *P* = 0.174). Furthermore, diabetes was not a predictor of major adverse events following CABG surgery (adjusted OR 1.35, 95% CI 0.86, 2.11; *P* = 0.188) **(**Table 2**).**

Diabetic patients experienced a higher proportion of postoperative arrhythmia than non-diabetic patients (47% vs. 44%, respectively). However, it was not statistically significant (crude OR 1.20, 95% CI 0.99–1.43, *P* = 0.052). While, after adjustment by age, gender, ethnicity, obesity, opium consumption, and smoking status, we found an association between diabetes and postoperative arrhythmia, as diabetes was associated with a 30% increase in the risk of postoperative arrhythmia (adjusted OR 1.30, 95% CI 1.08–1.57, *P* = 0.006) (Table 2).

**Fig. 3:**
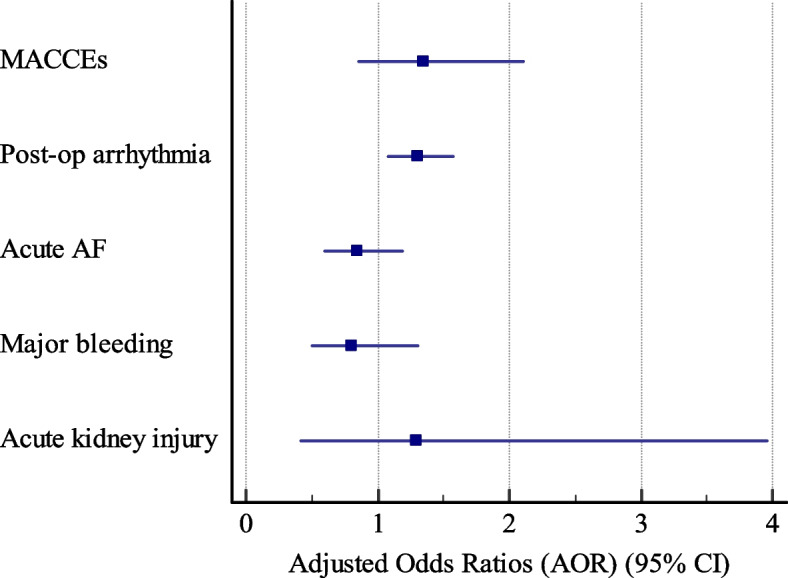
Adjusted odds ratios for study outcomes. MACCEs: Major adverse cardiac and cerebrovascular events (i.e., composite of MI, stroke and cardiovascular death). Post-op: Postoperative. AF: Atrial fibrillation

The proportions of acute AF, major bleeding, and AKI were not different between the two groups. Crude analyses indicated no association between diabetes and acute AF (Crude OR 0.80, 95% CI 0.60–1.1; *P* = 0.158), major bleeding (Crude OR 0.75, 95% CI 0.47–1.18; *P* = 0.216) or AKI (Crude OR 1.39, 95%CI 0.46, 4.15, *P* = 0.556) after surgery. Adjusted analyses indicated that diabetes was not a predictor of acute AF (adjusted OR 0.85, 95% CI 0.60–1.19, *P* = 0.340), major bleeding (adjusted OR 0.80, 95% CI 0.50–1.30, *P* = 0.636) or AKI (adjusted OR 1.29, 95% CI 0.42, 3.96, *P* = 0.656) after CABG surgery, as well (Table 2). The results of the adjusted analyses for study outcomes are demonstrated in Fig. 3.

## Discussion

In this retrospective cohort study of patients undergoing CABG surgery, patients with T2DM were significantly younger and had a higher percentage of women than other patients. Diabetic patients were treated with more preoperative medications, including Beta-blockers, Statins, and Aspirin. Furthermore, being burdened with DM was significantly associated with a higher chance of developing three-vessel disease and receiving more than three grafts during the surgery. However, even after adjustment for other known risk factors, DM was not a predictor of in-hospital major adverse events in these patients. On the other hand, the adjusted analysis indicated that DM was a predictor of postoperative arrhythmias and increased the risk by 30%.

In addition to being a major risk factor for CAD, diabetes can affect the treatment strategy for CAD [33]. Accordingly, 20 to 30% of patients undergoing CABG are burdened with diabetes [34–36].

In our study, diabetic patients undergoing CABG surgery were significantly younger than non-diabetic patients (76.5% under 65 years old vs. 70%, *P* = 0.003). These results were consistent with the Wang et al. study [37], in which the mean age of diabetic patients treated with Insulin who underwent CABG was significantly lower than non-diabetic patients [mean (SD): 62.7 (9.3) vs. 64.2 (8.4), *P* < 0.001]. In this regard, Stamou et al. [38] revealed that DM was more common among CABG patients aged 60–69 years compared to patients older than 80 (28% vs. 15%). In a cohort study conducted in the USA and Canada, Carson et al. [34] showed that diabetic patients treated with Insulin undergoing CABG are younger than non-diabetic patients [mean (SD): 63.8 (10.2) vs. 65.1(1.09)]; Also, consistent with our study, they revealed that male gender predominancy was imperceptible among DM patients (55.4% vs. 74.0% for non-diabetics).

Three-vessel disease and LMCA stenosis are indications for CABG surgery [39]. In a 2015 study, Jia et al. [40] utilized CT coronary angiography to compare CAD in patients with and without DM. They reported a significant association between DM and the number of diseased segments [OR (95%CI): 2.14 (1.09–2.6)] and a higher rate of multivessel disease among diabetic patients (15% vs. 7%, *P* < 0.001). Our study showed that three-vessel disease was significantly more common among diabetic patients (71.0% vs. 66.5, P: 0.020), but no significant difference was observed regarding the left main coronary artery (LMCA) stenosis (*P* = 0.153). This was consistent with the Yamaguchi et al. [41] study, in which three-vessel disease was significantly more prevalent among diabetic CABG candidates (75.1% vs. 68.5%, *P* < 0.001). Furthermore, in their study, the proportion of LMCA stenosis was not significantly different among the two groups. In 2020, wang et al. [37] conducted a study on 4325 CABG patients and reported that both three-vessel disease and LMCA stenosis were significantly more common among Insulin treated diabetic patients compared to non-diabetic patients. In this regard, no difference was detected among non-diabetic and oral-treated diabetic patients.

In Yamaguchi et al. study [41], there was no significant difference between diabetic and non-diabetic patients regarding post-CABG in-hospital death or stroke; Our study supports their findings, suggesting that DM is not significantly associated with in-hospital adverse events. Further to previous studies, DM was not a predictor of in-hospital MACCE following CABG surgery after adjustment by other risk factors. Furthermore, in a Chinese cohort study by Zhang et al. [42], DM was not associated with in-hospital post-CABG death, cerebrovascular accident (CVA), or MI. However, it was a predictor of long-term death or CVA. Their study assessed the resources and surgery costs in both groups, revealing that DM patients had higher costs for both initial hospitalization and follow-up.

Additionally, we analyzed the distribution of other postoperative complications in the two groups. After adjustment for potential confounders, our analysis showed that DM is a predictor of postoperative arrhythmias, increasing its risk by 30%. However, we detected no association between diabetes and postoperative AF. Diabetic patients are generally associated with a higher rate of arrhythmia [43–45]. However, studies have contradictory reports regarding diabetes and postoperative arrhythmias; In a retrospective cohort study on the assessment of postoperative Atrial Fibrillation (POAF) in CABG patients, Ismail et al. reported diabetes as an independent factor for POAF in these patients [46]. Mangi et al. also reported significantly higher rates of diabetes in post-CABG patients with POAF compared to patients without postoperative arrhythmia [47]. Although, in a meta-analysis on post-CABG arrhythmia by Woldendorp et al., diabetes was not significantly higher in patients with POAF compared to patients with a normal postoperative sinus rhythm [48].

One of the most serious postoperative complications in our patients was post-CABG major bleeding. The adjusted analysis indicated that diabetes was not associated with major postoperative bleeding in CABG patients. In a multicenter European study, Biancari et al. assessed the risk factors of post-CABG bleeding. Similar to our results, they indicated that diabetes was not associated with an increased risk of post-CABG bleeding [49]. Moreover, in the results of a large-scale study on post-CABG bleeding by Hansson et al., diabetes was also not associated with post-CABG bleeding [26].

Furthermore, our study failed to detect an association between diabetes and postoperative AKI. Previous studies have reported contradictive results. While various studies have indicated diabetes as a risk factor for postoperative AKI [50–52], several other studies did not suggest diabetes as a significant risk factor [53–55]. Inconsistencies in the definition of AKI might be a main contributing factor to the variations observed in the study results [56].

Due to its high prevalence and increasing incidence rate, diabetes is a significant public health issue in Iran [20, 57, 58]. Additionally, compared to previous years, the age-standardized mortality rate of DM had a notable rise in 2015 (11.3% in 2015 vs. 8.7% in 2000) [59]. In a 2011 study, Javanbakht et al. [60] reported 842.6 ± 102 USD for the average medical cost of diabetes per capita, and 412.8 ± 64.5 USD were complication costs. They reported cardiovascular disease expenditure as the most significant complication cost component (42.3%). Thus, conducting extensive studies on the issue in different regions is necessary to develop effective management strategies for cardiovascular disease in diabetic patients.

### Limitations

This large-scale study was conducted in Northeastern Iran. The current study was the first from this region, including ethnic diversities, especially Turkmen ethnicity. However, our study faced some limitations. Several variables, such as duration of diabetes, HbA1C level, and type of antidiabetic medications (oral pills or Insulin), were unavailable in the dataset we used to consider in the analysis, which may affect the study results. In this study, the in-hospital outcome was considered, and the long-term outcomes were not evaluated.

## Conclusion

To the best of our knowledge, this is the first study from Northeastern Iran focusing on the effect of DM on the in-hospital MACCEs and postoperative complications after CABG surgery. Severe and multivessel coronary artery disease was more common among diabetic CABG patients compared to other CABG patients. This difference could explain the higher number of grafts used in these patients during the operation. Despite the differences, the risk of in-hospital MACCEs, acute AF, major bleeding, and AKI was not statistically significant between diabetic and non-diabetic patients following a CABG surgery. While, diabetes was associated with a 30% increase in the risk of postoperative arrhythmia, which requires further attention.

## Supplementary Information

**Additional file 1: Appendix**. **Table S1.** Comparison of In-hospital MACCEs (+Acute kidney injury) between patients with and without diabetes mellitus undergoing isolated on-pump CABG surgery.

## Data Availability

The datasets regarding the current study are available from the corresponding author upon reasonable request.

## Abbreviations

DM: Diabetes mellitus
CABG: Coronary artery bypass graft
T2DM: Type 2 diabetes mellitus
MACCEs: Major adverse cardiac and cerebrovascular events
FPG: Fasting plasma glucose
AKI: Acute kidney injury
SD: Standard deviation
PCI: Percutaneous interventions
CAD: Coronary artery disease
HTN: Hypertension
REC: Research ethics committee
BMI: Body mass index
CDC: Center for disease control and prevention
MI: Myocardial infarction
COPD: Chronic obstructive pulmonary disease
LDL: Low-density lipoprotein
HDL: High-density lipoprotein
BB: Beta-blocker
LVEF: Left ventricular ejection fraction
TTE: Transthoracic echocardiography
LMCA: Left main coronary artery
CAG: Coronary artery angiography
LIMA: Left internal mammary artery
IQR: Interquartile range
CIE: Change-in-estimate
CPB: Cardio pulmonary bypass
CI: Confidence interval
CT: Computed tomography
ICU: Intensive care unit
POAF: Post operative atrial fibrilation
