# Supplementary material for: Type 2 diabetes mellitus and In-hospital Major Adverse Cardiac and Cerebrovascular Events (MACCEs) and postoperative complications among patients undergoing on-pump isolated coronary artery bypass surgery in Northeastern Iran
Source: BMC Cardiovasc Disord. 2023 Mar 11;23:130. doi: 10.1186/s12872-023-03163-5 (PMC10007752; doi:10.1186/s12872-023-03163-5)
Supplement: Supplementary file 1 — Additional file 1: Appendix. Table S1. Comparison of In-hospital MACCEs (+Acute kidney injury) between patients with and without diabetes mellitus undergoing isolated on-pump CABG surgery. [file 12872_2023_3163_MOESM1_ESM.docx]

**Appendix:**

Considering the variations in study settings and populations between the existing literature, the definition of in-hospital MACEs is also not similar between these studies. However, most studies considered three components of MI, stroke, and death as in-hospital MACCEs. Thus, we used the same variables as in-hospital MACE (**Table 2**). Subsequently, we assessed acute kidney injury as an additional component (**Table S1**). According to this table, DM was not associated with in-hospital adverse events following CABG, even when we considered acute kidney injury as one of its components.

| **Table S1**. Comparison of In-hospital MACCEs (+Acute kidney injury) between patients with and without diabetes mellitus undergoing isolated on-pump CABG surgery | | | | | | | |
| --- | --- | --- | --- | --- | --- | --- | --- |
| **In- hospital outcomes** | | **Diabetes Group** | | **Crude OR**  **( 95 % CI)** | **P_value** | **Adjusted OR**  **( 95% CI)** | **P_value** |
|  |  | **Without DM**  **(n=1062)** | **With DM**  **(n=894)** |  |  |  |  |
| **MACCEs**, n (%) | |  |  | 1.34 (0.88, 2.05) | 0.169 | 1.31 (0.85, 2.02) | 0.214‡ |
|  | Yes | 43 (4.0) | 48 ( 5.0) |  |  |  |  |
|  | No | 1019 ( 96.0) | 846 (95.0) |  |  |  |  |
| OR: Odds ratio; CI: Confidence Interval; P_value: Probability value  MACCEs: major adverse cerebrocardiovascular events ( i.e., composite of MI, Stroke, cardiovascular death, and acute kidney injury)  AF: Atrial fibrillation  **Statistically significant (P< 0.05)  ‡ Adjusted by age ≥65 years, sex, ethnicity, obesity, opium consumption, and smoking | | | | | | | |
